# RET rearrangement as a mechanism of resistance to ALK-TKI in non-small cell lung cancer patient with EML4-ALK fusion: A case report

**DOI:** 10.1016/j.heliyon.2024.e29928

**Published:** 2024-04-22

**Authors:** Huan Yan, Liang Zeng, Yongchang Zhang

**Affiliations:** a Graduate Collaborative Training Base of Hunan Cancer Hospital, Hengyang Medical School, University of South China, Hengyang, Hunan, 421001, China; b Department of Medical Oncology, Lung Cancer and Gastrointestinal Unit, Hunan Cancer Hospital/The Affiliated Cancer Hospital of Xiangya School of Medicine, Central South University, Changsha, 410013, China

**Keywords:** RET-Rearranged, EML4-ALK, Dual-targeted treatment, NSCLC, Case report

## Abstract

Patients with non-small cell lung cancer (NSCLC) and anaplastic lymphoma kinase (ALK) mutations have previously derived substantial benefits from ALK tyrosine kinase inhibitors (ALK-TKIs). However, resistance may develop in some patients. We present a case of co-mutation with anaplastic lymphoma kinase (ALK) and rearranged during transfection (RET)-rearranged NSCLC, representing a novel resistance mechanism to ALK-TKIs, in which the patient exhibited a favorable response to combination therapy with ensartinib and pralsetinib. Notably, the patient survived 12 months without experiencing adverse events, a rare occurrence in ALK-rearranged lung adenocarcinoma cases. This case provides further evidence for the existence of RET rearrangements in ALK-positive lung cancer and their potential treatment response to a combination of ALK inhibitors and pralsetinib. This case underscores that a dual-target therapy involving ALK inhibitors, specifically ensartinib and pralsetinib, could be a viable approach in cases of RET-rearranged lung cancer with concurrent targetable ALK mutations. We propose the consideration of this dual-target approach, specifically employing ensartinib and pralsetinib, in managing RET-rearranged lung cancer coexisting with targetable ALK mutations. Given the potential efficacy of these treatments, it is imperative to proactively conduct molecular profiling tests in NSCLC patients upon the emergence of resistance.

## Highlights

• NCOA4-RET is an acquired resistance mechanism in ALK-fusion NSCLC.
• Ensartinib and pralsetinib exhibited robust activity in a patient with NCOA4-RET.
• Re-biopsy and gene testing are crucial for developing personalized treatment strategies.

## 1 Introduction

Approximately 5–7% patients with NSCLC develop ALK fusion, with over 20 variants identified, and EML4-ALK being the most prevalent [1,2]. Although targeted therapy has significantly improved patient survival, resistance remains unavoidable [[3], [4], [5]]. ALK kinase domain mutations, bypass activation, tissue-type transformation are among the confirmed resistance mechanisms, together with other unknown mechanisms [[6], [7], [8], [9], [10]].

RET rearrangements have been found as a resistance mechanism to epidermal growth factor receptor tyrosine kinase inhibitors (EGFR-TKIs) in EGFR-mutant NSCLC patients [[11], [12], [13], [14]]. However, to date, no instances of a newly identified bypass-activated mechanism of resistance to ALK-TKIs have been reported.

Herein, the patient developed secondary RET fusion after resistance to ALK-TKIs and was treated with a combination of ensartinib and pralsetinib dual-target therapy. This, to the best of our knowledge, is the first report to show that there can be reversal of resistance and provides a clinical evidence-based medical suggestion for viability of combination therapy.

## 2 Case presentation

A 61-year-old nonsmoking woman underwent video-assisted thoracoscopic surgery involving left upper lobe resection and lymph node dissection under general anesthesia on November 13, 2017. Postoperative pathology results revealed adenocarcinoma, and immunohistochemistry results were positive for TTF-1, Napsin A, CK7, CK-P, and ALK, but negative for Syn and CK5/6. A 37-gene next-generation sequencing (NGS) panel (OrigiMed, Shanghai, China) of tumor DNA identified an EML4-ALK fusion (V3) with a TP53 c.376-1G > A mutation. The patient underwent R2 resection, and the postoperative stage was pT1cN2M0 IIIA. Subsequently, the patient received treatment with a combination of pemetrexed and cisplatin for four cycles and adjuvant radiotherapy for the residual lesion and draining lymph node area, with a total dose of 95%GTV 64 Gy/2.13 Gy/30f, 95%PTV 54 Gy/1.80 Gy/30f.

**Fig. 1:**
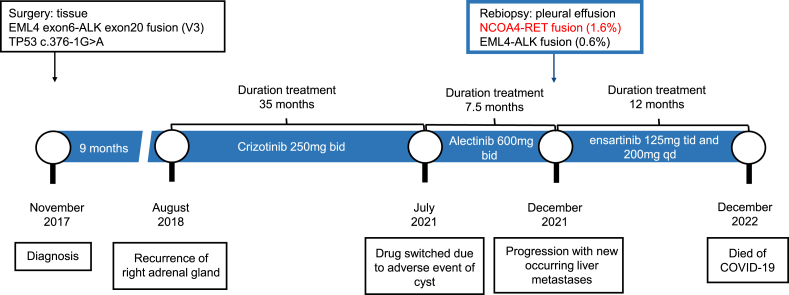
Clinical timeline summarizing the different therapeutic sequences, the sites of relapses and the different moments of disease progression.

**Fig. 2:**
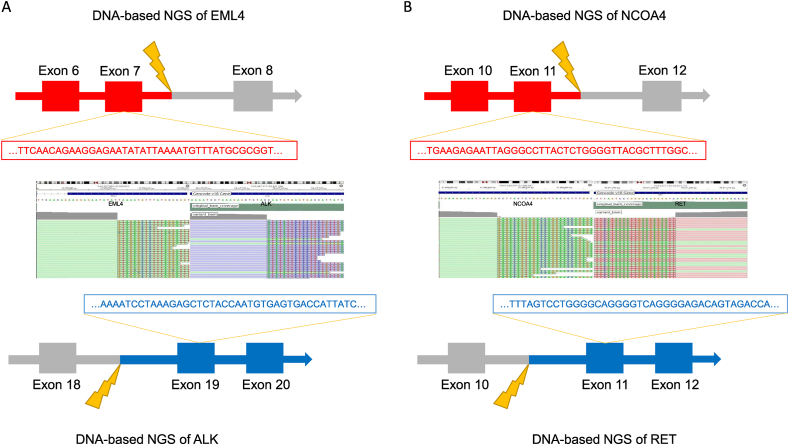
Gene fusion of EML4-ALK and RET. The left panel (A) illustrates the EML4-ALK fusion, where the fusion occurs between EML4 exon 7 and ALK exon 19, resulting in a single fused gene. The right panel (B) depicts the NCOA4-RET fusion, where the fusion arises from NCOA4 exon 11 and RET exon 11, generating a fused gene sequence.

In August 2018, re-examination using positron emission tomography-computed tomography (PET-CT) showed a soft tissue nodule on the right adrenal gland with an SUV of 6.8, which was indicative of disease recurrence. Treatment with crizotinib 250 mg bid was initiated, and approximately 2 months later, the patient achieved a partial response (PR) with shrunken right adrenal metastasis. The patient developed bilateral renal cysts during 35 months of crizotinib treatment, which persisted and continued to enlarge (Fig. 1 and Fig. S1). Considering the rare adverse reaction profile of crizotinib and the patient's lack of history of renal cysts, it was deemed appropriate to discontinue crizotinib and switch to alectinib 600 mg bid [15] in July 2021. The bilateral renal cysts gradually improved, but the patient repeatedly showed a grade 3 increase in transaminase and bilirubin levels. After 7.5 months of treatment with alectinib, the patient experienced pleural effusion and widespread liver metastases. Thoracentesis revealed the presence of cancer cells in the pleural effusion (Fig. 1). Molecular testing using NGS (1021-gene panel, Geneplus, Beijing, China) demonstrated NCOA4-RET and EML4-ALK fusion (non-V1/V3). The allele frequency of the NCOA4-RET fusion was 1.6 %, compared to 0.6 % for the EML4-ALK fusion (non-V1/V3) (Fig. 2).

**Fig. 3:**
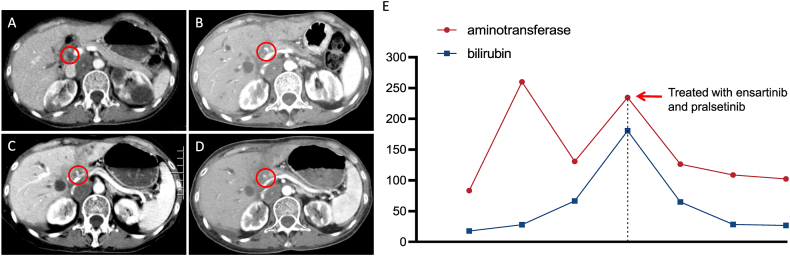
(A) CT scan of the patient's normal bile duct at baseline with contrast (large red circles). (B) CT scan at the onset of biliary dilatation (red circles). (C–D) Serial CT scans showed changes in biliary dilatation treated with pralsetinib combined with ensartinib at 2 and 4 months, respectively (red circles). (E) Serum levels of bilirubin and aminotransferase decreased and returned to the normal range within two months after switching to pralsetinib combined with ensartinib (red arrows). (For interpretation of the references to color in this figure legend, the reader is referred to the Web version of this article.)

Subsequently, the patient presented with jaundice and yellow skin discoloration. A follow-up liver function test indicated elevated levels of alanine aminotransferase at 188 U/L and total bilirubin at 180.8 μmol/L (Fig. 3E). Compared to previous radiological findings, bile duct dilatation seemed to progress, and the treatment was switched to ensartinib 125 mg tid combined with pralsetinib 200 mg qd on January 1, 2022 (Fig. 3A–D). After two months, bilirubin levels decreased to 26.9 μmol/L, alanine aminotransferase decreased to 102.6 U/L, and the pupil and skin yellowing returned to normal. CT re-examination showed improved bile duct dilatation and absorbed chest fluid with PR. Unfortunately, on December 26, 2022, the patient died of coronavirus disease (COVID-19). Until death, the patient received dual-targeted treatment with ensartinib combined with pralsetinib for more than 12 months, with sustained benefits, safety, tolerability, and no other adverse events.

Written informed consent was obtained from the patient for publication of this case report and the use of accompanying images.

## 3 Discussion

This case report emphasizes the vital role of molecular testing in the identification of resistance mechanisms in lung cancer. Concurrent driver gene alterations can offer valuable clinical insights for treatment decisions and prognosis [16]. Based on the information available, this case represents the first report of lung adenocarcinoma harboring NCOA4-RET fusion as a resistance mechanism to ALK-TKI, with simultaneous use of targeted therapy against both drivers. In this patient, the treatment options primarily revolved around combining chemotherapy with known oncogenic drivers or continuing combination therapy with targeted therapy. Current research suggests that combination therapy with later-line chemotherapy and TKI may not improve the outcome [17], but this remains an area of exploration in the treatment of brain metastasis with TKIs.

Due to the initial response to systemic therapy and the patient's condition, the tumor biopsy tissue was not from the same site, and it was not possible to determine whether the two mutations were derived from the same clone. Tumor heterogeneity is the primary limitation of our study [18]. In a similar situation in the future, we can consider obtaining samples for single-cell sequencing or simultaneously conduct RET fluorescence in situ hybridization and Ventana ALK (D5F3) detection. These approaches would enable us to determine whether the mutations originated in the same cell line. Co-occurring drivers are uncommon owing to the apparent mutual exclusion of oncogenic solid drivers in NSCLC. Nonetheless, following application of NGS detection in the clinic, our understanding of the NSCLC genomic map has gradually deepened [16]. In a retrospective study of 3077 NSCLCs, only 46 patients had co-occurring and potentially targetable carcinogenic drivers, including EGFR, ALK, and MET, as defined by NGS testing.

In the ARROW trial, a single-arm, multicenter, non-randomized, open-label, multi-cohort study in patients with RET fusion-positive NSCLC and other advanced solid tumors, the standard dose of pralsetinib was 400 mg/day [19]. However, in this case, because of a significant increase in bilirubin, the dose of pralsetinib was reduced to 200 mg/day in combination therapy. After treatment with pralsetinib 200 mg qid combined with ensartinib 125 mg tid, the patient's alanine aminotransferase and bilirubin levels rapidly decreased, suggesting that the treatment was effective.

This case report presents the first documented occurrence of RET fusion in a patient with EML4-ALK-positive NSCLC following acquired resistance to ALK-TKI therapy. The patient successfully responded to combination therapy with pralsetinib and ensartinib, effectively overcoming acquired resistance. This significant research outcome has valuable implications and can serve as a reference for patients with similar circumstances. The dual-targeted treatment approach involving ensartinib combined with pralsetinib appears promising as an effective treatment method. However, further research is required to validate this conclusion and to provide more robust evidence.

## 4 Conclusion

Herein, we report the first documented case of RET rearrangement as a novel acquired resistance mechanism in a patient with EML4-ALK mutated NSCLC. Remarkably, this case substantiates that the treatment with ensartinib and pralsetinib was sustained for over 12 months without any adverse events. These findings are promising and offer valuable insights for patients with similar conditions.

## Funding statement

This work was financially supported by the 10.13039/501100004735Natural Science Foundation of Hunan Province (grant numbers: 2021SK51105、2021RC3118、2023JJ30368).

## Data availability statement

Data included in article/supp. material/referenced in article.

## Patient consent

Informed consent was obtained from the patient.

## Additional information

No additional information is available for this paper.

## CRediT authorship contribution statement

**Huan Yan:** Writing – review & editing, Writing – original draft, Validation, Supervision, Software, Methodology, Investigation, Data curation. **Liang Zeng:** Writing – review & editing, Visualization, Validation, Methodology, Funding acquisition, Formal analysis, Data curation. **Yongchang Zhang:** Writing – review & editing, Visualization, Supervision, Project administration, Funding acquisition, Conceptualization.

## Declaration of competing interest

The authors declare that they have no known competing financial interests or personal relationships that could have appeared to influence the work reported in this paper.
