# Supplementary material for: RET rearrangement as a mechanism of resistance to ALK-TKI in non-small cell lung cancer patient with EML4-ALK fusion: A case report
Source: Heliyon. 2024 Apr 22;10(9):e29928. doi: 10.1016/j.heliyon.2024.e29928 (PMC11064130; doi:10.1016/j.heliyon.2024.e29928)
Supplement: Multimedia component 1 [file mmc1.pptx]

## Slide 1
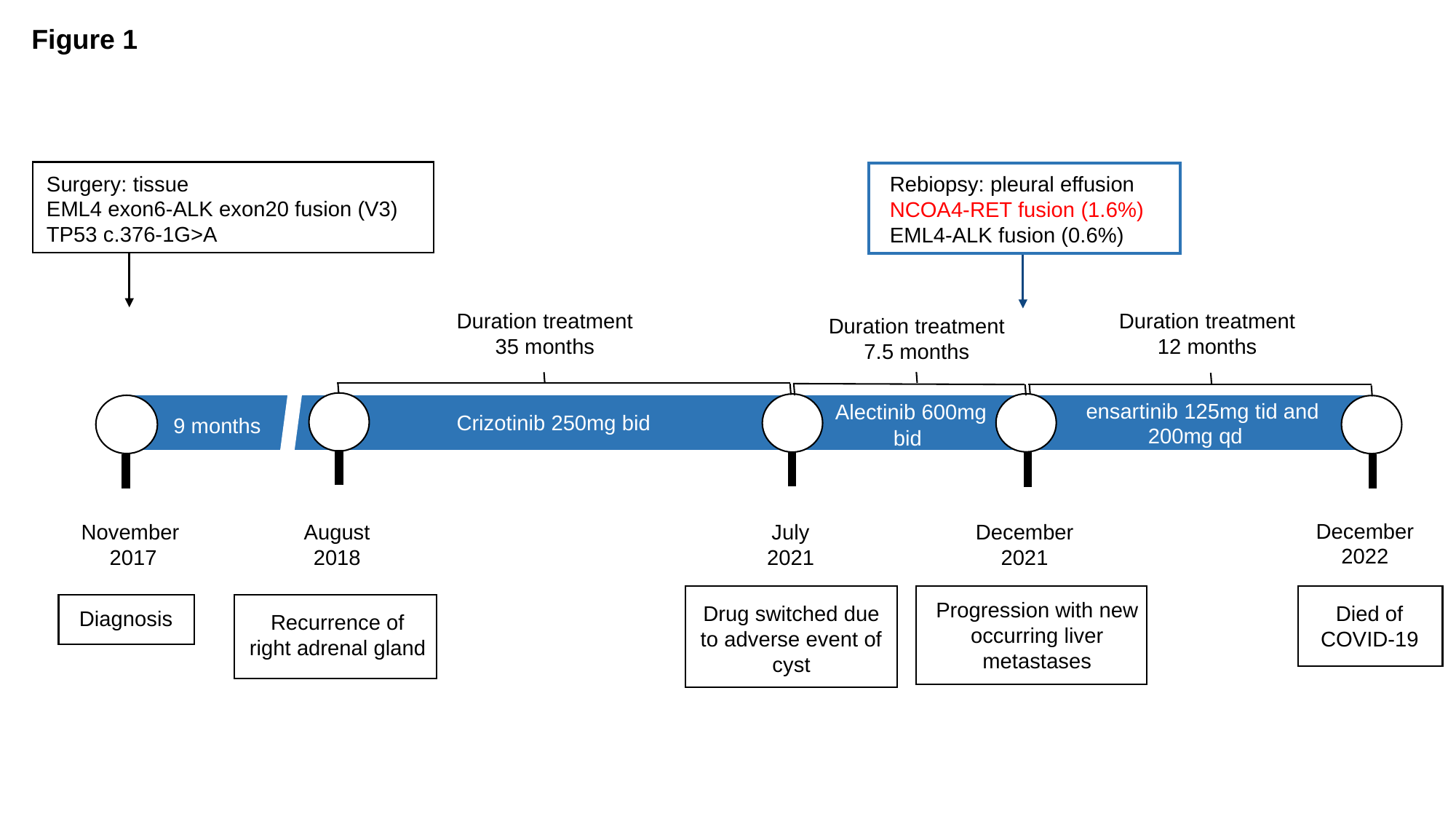

Figure 1
Surgery: tissue
EML4 exon6-ALK exon20 fusion (V3)
TP53 c.376-1G>A
Rebiopsy: pleural effusion
NCOA4-RET fusion (1.6%)
EML4-ALK fusion (0.6%)
Duration treatment 35 months
Duration treatment 12 months
Duration treatment 7.5 months
 Alectinib 600mg bid
ensartinib 125mg tid and 200mg qd
Crizotinib 250mg bid
9 months
December 2022
November
2017
August 2018
July 2021
December 2021
Progression with new occurring liver metastases
Drug switched due to adverse event of cyst
Died of COVID-19
Diagnosis
Recurrence of right adrenal gland

## Slide 2
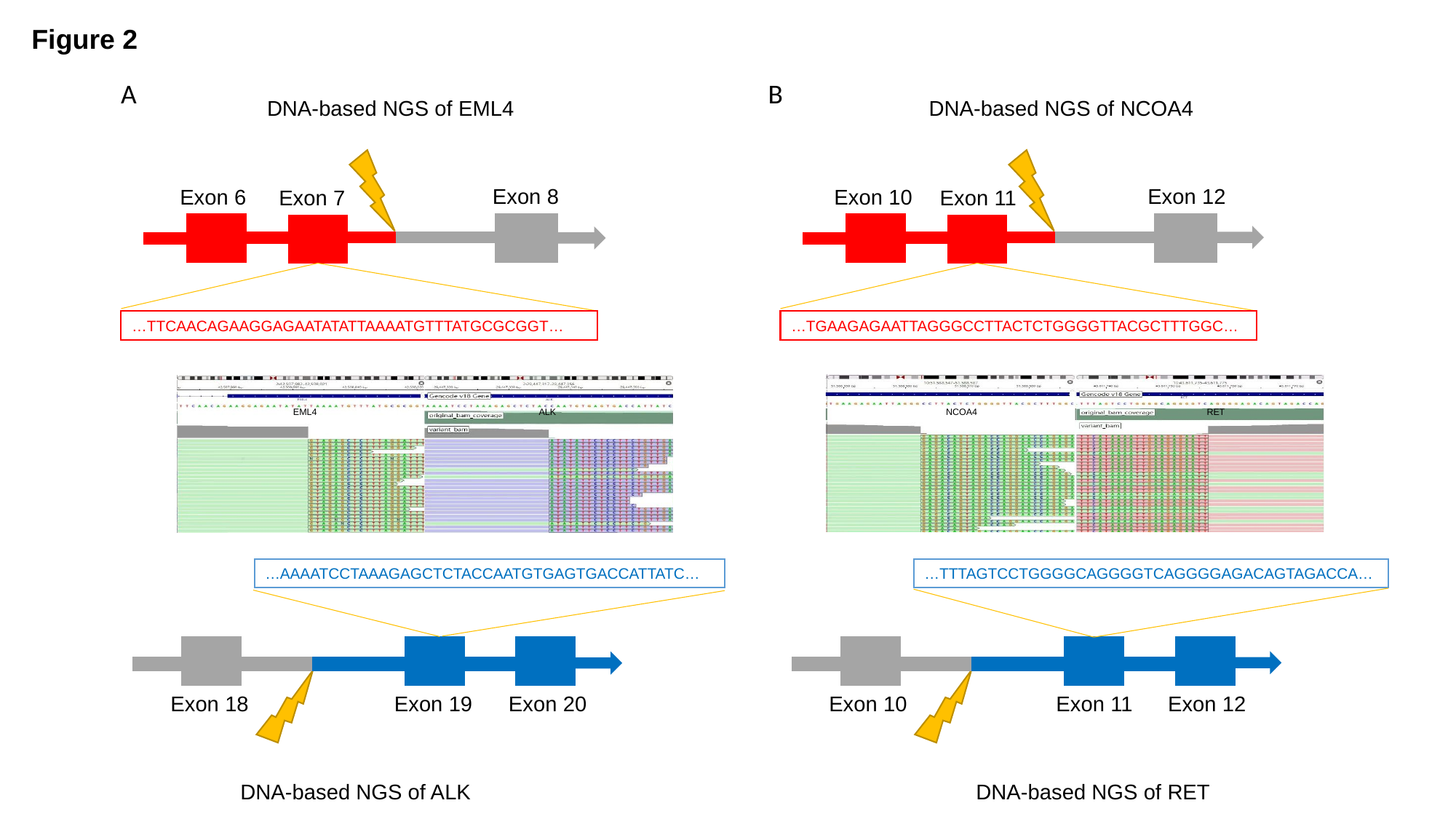

Figure 2
A
B
DNA-based NGS of NCOA4
Exon 12
Exon 10
Exon 11
…TGAAGAGAATTAGGGCCTTACTCTGGGGTTACGCTTTGGC…
NCOA4
RET
…TTTAGTCCTGGGGCAGGGGTCAGGGGAGACAGTAGACCA…
Exon 10
Exon 12
Exon 11
DNA-based NGS of RET
DNA-based NGS of EML4
Exon 8
Exon 6
Exon 7
…TTCAACAGAAGGAGAATATATTAAAATGTTTATGCGCGGT…
EML4
ALK
…AAAATCCTAAAGAGCTCTACCAATGTGAGTGACCATTATC…
Exon 18
Exon 19
Exon 20
DNA-based NGS of ALK

## Slide 3
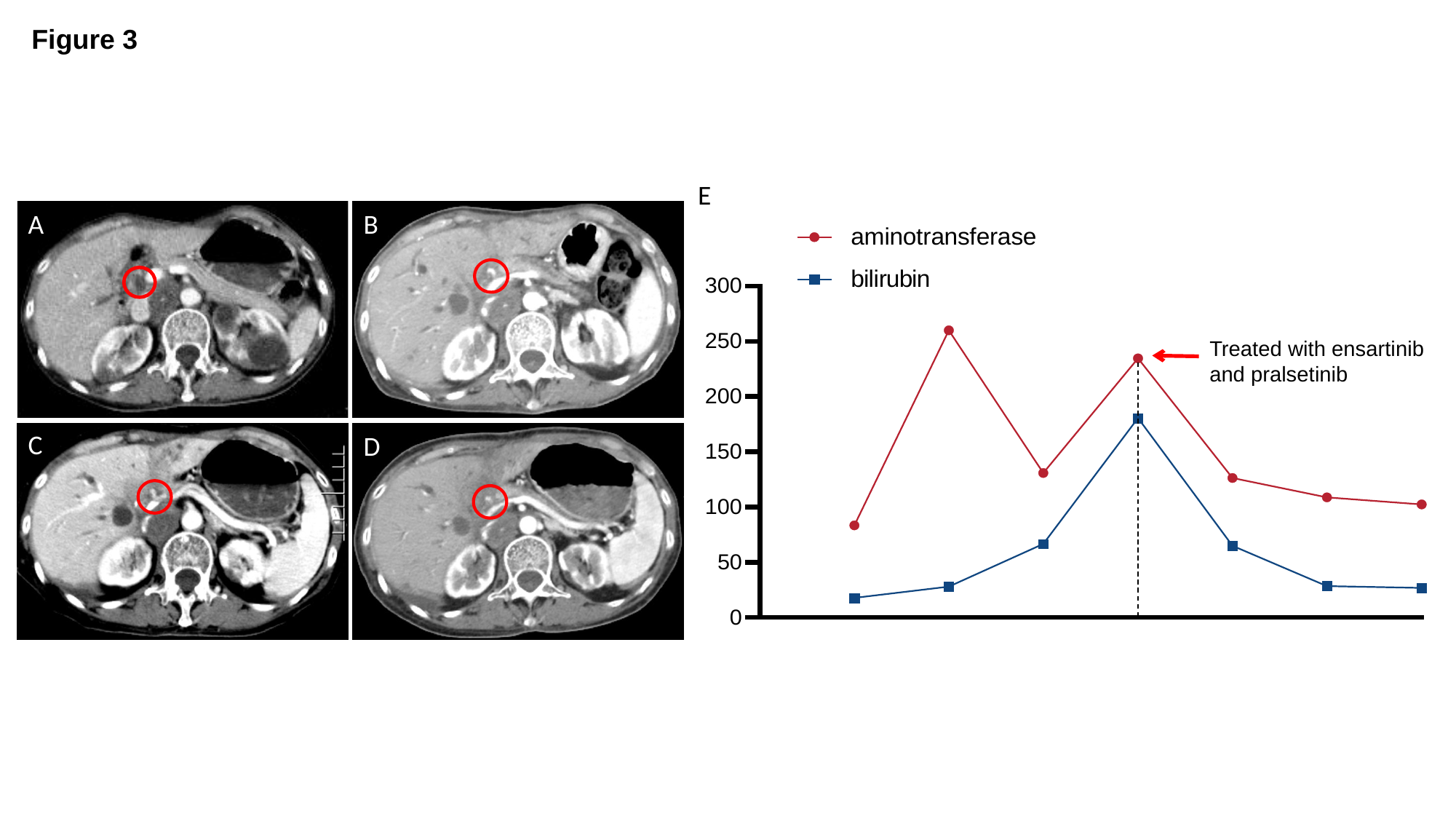

Figure 3
E
A
A
A
B
Treated with ensartinib and pralsetinib
C
D

## Slide 4
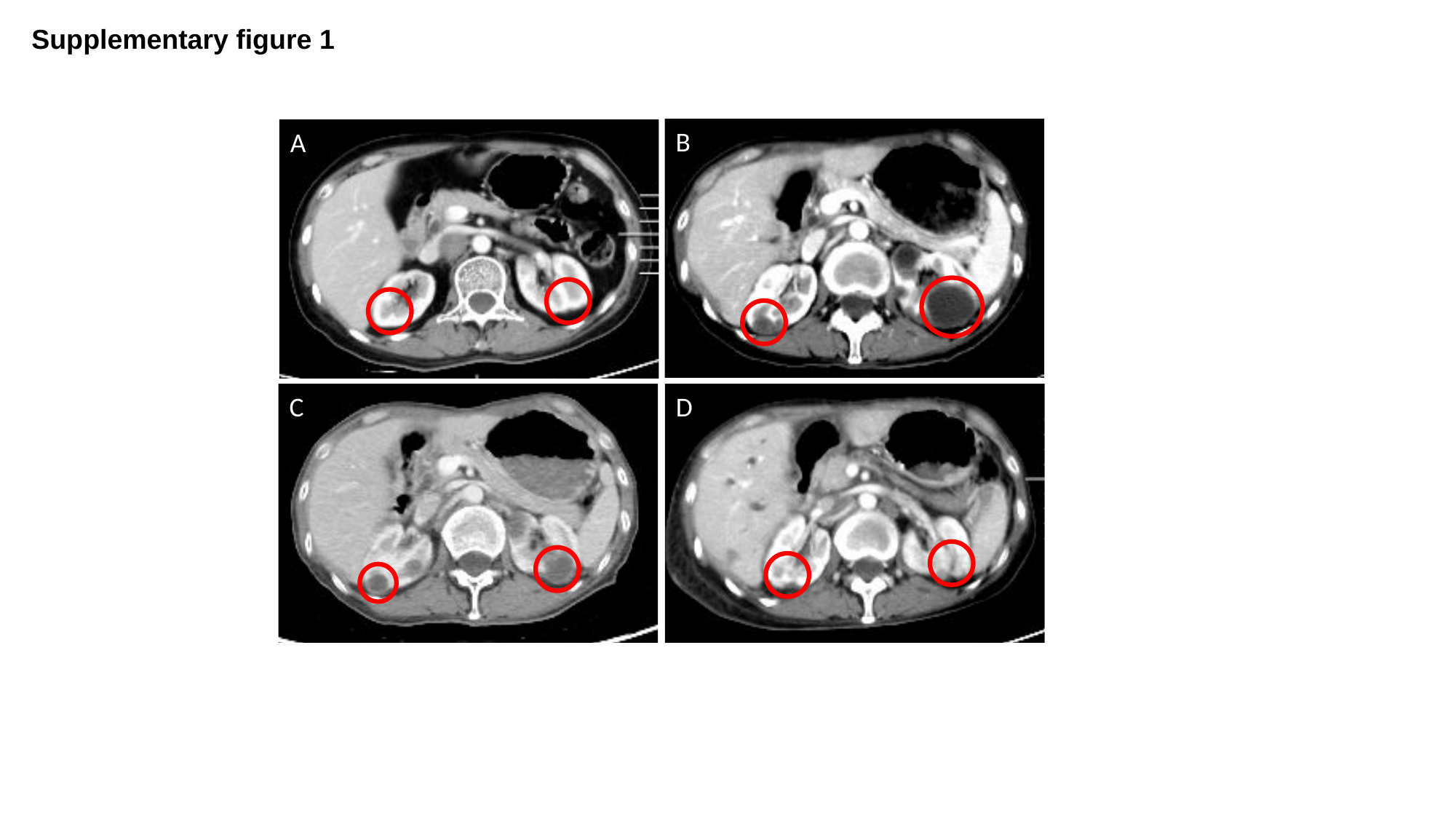

Supplementary figure 1
B
A
C
D
